# The Power of Research and Stakeholder Collaboration in Enhancing Early Childhood Care and Education in Low- and Middle-Income Countries

**DOI:** 10.3390/children11060698

**Published:** 2024-06-07

**Authors:** Nirmala Rao, Caroline Cohrssen, Manya Bala

**Affiliations:** 1 Faculty of Education, The University of Hong Kong, Hong Kong, China; 2 School of Education, University of New England, Armidale, NSW 2350, Australia

The early years of a child’s life are crucial in laying the foundations of learning and development that shape life trajectories. Research across various fields, from neuroscience to education and economics, demonstrates the benefits of children participating in quality early childhood care and education (ECCE) programs. Quality early learning fosters optimal outcomes across developmental domains, but also educational achievement, societal integration, health, and labour market outcomes [1,2]. Conversely, negative experiences and exposure to inadequate ECCE have been shown to impede neurodevelopment, exerting a detrimental influence on learning and development, and ultimately may contribute to adverse long-term health, social, and economic consequences [3,4]. This comprehensive body of research highlights both the incentive and urgency to invest in ECCE. Indeed, Sustainable Development Goal 4.2 aims to “Ensure all girls and boys have access to quality early childhood development, care, and pre-primary education” and signifies the United Nations member states’ commitment to achieve this goal by 2030 [5]. During the 2022 World Conference of Early Childhood Care and Education, member states’ commitments to achieving SDG 4.2 were reaffirmed and, through the Tashkent Declaration, set out guiding principles to achieve this goal with a focus on innovation for transformation, strengthening policy, governance, and finance, and bolstering the ECCE workforce [6].

Extant research has demonstrated early childhood development to be a complex process that involves the dynamic interplay of a child’s biology and their environment. Consequently, great emphasis is placed on taking a holistic and ‘whole-of-child’ approach in ECCE when considering stakeholders’ roles and opportunities to support child development and to bridge gaps in ECCE [7]. Here, stakeholders include caregivers, parents, governments, researchers, and the ECCE workforce. The Nurturing Care Framework was ground-breaking in providing an empirically supported framework to guide the development and implementation of ECCE policies, services, and interventions that are holistic [8]. The Nurturing Care Framework underscores the importance of collaboration among stakeholders in promoting optimal early childhood development. Partnerships and shared resources enable effective implementation of policies and interventions and contribute to improved outcomes for children. This Special Issue on ECCE in low- and middle-income countries (LMICs) is a collection of case studies that reflect many of the necessary advancements set out in both the Tashkent Declaration and the Nurturing Care Framework. They demonstrate ways in which data and resources have been leveraged in diverse country contexts as well as collaborations with stakeholders to generate an evidence base for policy, intervention, and effective monitoring relating to ECCE.

Globally, important progress has been made across indicators of SDG 4.2. The Gross Enrolment Ratio (GER) of children attending pre-primary education increased from 46% in 2010 to 61% in 2020 [9]. As of 2021, 99 countries had early childhood development policies or action plans, compared with 76 countries in 2019 [10,11]. Despite substantial efforts to enhance ECCE provision globally, stark demographic disparities between and within countries are apparent due to persisting socio-economic inequalities. Between-country income levels constitute a consistent disparity. For example, there was a 12.6% gap in early childhood education GERs between upper-middle-income countries and LMICs in 2020 [7]. Additionally, 61.8% of children were reported to be living in a stimulating home environment in LMICs compared with 85.3% in upper-middle countries [12].

Indeed, LMICs experience numerous obstacles to equity in access to quality early childhood education. These include limited financial resources, poor infrastructure, gaps between policy and implementation, and significant workforce shortages. These impact ECCE provision and participation and result in gaps in children’s learning and development, both within and between countries. Recent estimates using the Early Childhood Development Index found that 22.5% of children in LMICs were found to have developmental delays [13]. This is particularly worrying, since globally, most children under five years of age live in LMICs. To gain a better understanding of existing gaps not only on children’s developmental and educational status but also on ECCE service quality, effective data collection and monitoring is essential [14]. However, many of the existing assessment and monitoring tools are developed in Western, Educated, Industrialised, Rich, and Democratic (WEIRD) contexts, which are not necessarily culturally or contextually relevant or applicable in LMICs [15]. Considering the aforementioned concerns, this timely Special Issue addresses the gaps and challenges prevalent in LMICs and contributes to paving the way forward in supporting and promoting equity in access to quality education for all.

Aboud and colleagues (Contributions 1 and 4) have contributed two papers to this Special Issue. Both report on case studies of a programme aimed at parents of children aged from birth to 36 months in Bhutan and Zambia. The first paper outlines how existing research on the implementation of the programme was used to inform and tailor a scaled-up programme. Their second paper focuses on the workforce delivering the parenting programme, specifically their training, workload, and quality of delivery. The research illustrates the collaborative approach called for in the Nurturing Care Framework and highlights its utility in improving the quality of ECCE delivery. Two papers provide comprehensive overviews of ECCE policy history, national context, existing inequities in ECCE, policy gaps, and subsequent policy recommendations. One does so for China by providing a chronological overview of the government’s efforts to provide equitable access to quality education from 2010 onwards (Contribution 2). Raikes and colleagues examine the economic and political contexts of ECCE in Brazil from the perspective of children’s rights (Contribution 5). Using different perspectives, both papers highlight the importance of carefully examining relevant contextual factors in the inequities of ECCE to develop meaningful, evidence-based recommendations. Giese and colleagues’ paper outlines the development of an early learning measurement tool to assess preschool quality and child outcomes in South Africa (Contribution 3). The paper comprehensively details the processes necessary to develop a culturally and contextually relevant measurement tool that addresses gaps in data quality and monitoring in LMICs.

Overall, the articles in this Special Issue highlight critical considerations to be considered when undertaking collaborative research in ECCE that is sensitive to the cultural context in which it is undertaken. This is essential for advancing understanding and improving ECCE and facilitates the exchange of knowledge, ideas, and best practices across different areas of ECCE. Furthermore, it promotes innovation, informs evidence-based policymaking, and ultimately improves access to and participation in high-quality education for all children.
